# Green Modification of Corn Stalk Lignin and Preparation of Environmentally Friendly Lignin-Based Wood Adhesive

**DOI:** 10.3390/polym10060631

**Published:** 2018-06-07

**Authors:** Sen Wang, Yalan Yu, Mingwei Di

**Affiliations:** College of Materials Science and Engineering, Northeast Forestry University, Harbin 150040, China; ws920303@163.com (S.W.); jhs4tt@naver.com (Y.Y.)

**Keywords:** corn stalk lignin, glyoxal, modification, adhesive, plywood

## Abstract

In this study, corn stalk lignin was used to react with non-volatile and non-toxic glyoxal under the catalysis of a sodium hydroxide solution, and a wood adhesive based on glyoxalated corn stalk lignin was prepared. The effect of the hydroxylation reaction on the structure and properties of lignin were studied using Fourier transform infrared spectroscopy (FTIR), ultraviolet spectrophotometry (UV), thermogravimetric analysis (TGA), titration tests, gel permeation chromatography (GPC), and differential scanning calorimetry (DSC). Compared with unmodified lignin, the glyoxalated corn stalk lignin had a significant improvement in hydroxyl content, activation, and thermal stability. At the same time, results from the GPC showed that the molecular weight increased compared with original corn stalk, possibly due to the secondary polycondensation reaction between lignin and glyoxal. Lignin-based environmental wood adhesives were prepared by mixing modified lignin and epichlorohydrin (ECH), and the dry strength of plywood reached 1.58 MPa. The mechanical strength and water resistance of plywood was improved significantly by mixing some aqueous emulsion into lignin-based adhesives, e.g., polyacrylic ester (AE) emulsion and aqueous polyurethane (PU) emulsion.

## 1. Introduction

Traditional fossil-fuel-based wood adhesives, e.g., urea-formaldehyde resin and phenol-formaldehyde resin, have been widely used in wood-based panel industry since the 20th century, due to their own high performance–price ratio [1]. Although some of these adhesives have already won the recognition of the market, there were some shortcomings and hidden dangers that have appeared gradually to this day, such as the decrease of raw materials, as well as environmental and health concerns. All of these problems intensified with the growing interest in environment-friendly biomass-based wood adhesives. Thus, some biomass resources have become the focus of research in recent years, such as starch [2,3,4], soybean protein [5,6,7,8], tannin [9,10,11], and lignin [12,13,14,15]. Lignin is the second-most abundant renewable resource in nature, only behind cellulose [16]. Lignin is a natural polymer with a large number of aromatic groups, which is made up of three monomers, namely syringyl (S), guaiacyl (G), and hydroxyl-phenyl (H) units, linked by a multitude of chemical bonds, ether linkages, and C–C linkages [17,18,19]. In contrast to the great number of literature [12,13,14,15], the way of industrial application for lignin-based wood adhesive is still poor. Most industrialization attempts in this field were not really achieved, due to some availability reasons from the technique, both process and economic.

The lignin from the byproduct of the pulping, papermaking, and bioethanol processes have already been tried for the preparation of wood adhesives in wood panel industry, mainly according to the following three different approaches. First, the pulping lignin liquors were directly blended into wood adhesives [20]; lignin is used just as filler in the adhesives in this process. With the introduction of lignin, both mechanical and thermostability properties of the adhesive show remarkable improvement, but only a small amount of lignin could blend into the adhesives, and the active groups on the lignin are useless in this system. The second way is based on the modified lignin from the methods of phenolization, hydroxylation, or laccase treatments, among others [21,22,23]. Today, most research focuses on how to substitute the modified lignin for part of petroleum-based components in wood adhesives. In order to ensure the performance of the adhesive, the addition of the modified lignin should not be too large. Furthermore, the problem has not been solved regarding the volatility of small toxic molecules, such as formaldehyde or phenol, in the process of preparation and application of adhesives. The third method is based on the reaction between environmentally friendly curing agents, such as polyisocyanates and epoxy, and the modified lignin [24,25]. Lignin acts as a reactive component in this way, and the active functional groups in lignin are fully utilized too. Until now, this approach is still the main method of preparing environmentally friendly lignin-based wood adhesives. 

Lignin can be used to react with formaldehyde under the catalysis of sodium hydroxide solution, while at the same time the hydroxymethyl (–CH_2_OH) groups can be introduced onto the lignin molecule during this process [26,27]. Recently, a new idea was proposed by the group of Pizzi [28], where they substituted glyoxal for formaldehyde in the hydroxylation reaction. Compared with formaldehyde, glyoxal has many advantages, e.g., the toxicity (LD50 rat ≥ 2960 mg/kg; LD50 mouse ≥ 1280 mg/kg) is far less than formaldehyde [28], it is non-volatile, and most of them can obtained from natural sources. Nevertheless, the reactivity of glyoxal is lower than formaldehyde, and it has been proved that there is no obvious influence on the property of adhesives using hydroxyethylated lignin compared with hydroxymethylated lignin [29].

Recently enzymatic hydrolysis lignin (EHL) has received considerable attention. Compared with the traditional industrial lignin, such as sulfonate lignin and alkali lignin, EHL is not treated by an alkaline medium or high temperature, which completely reserves the original structure and many functional groups, such as phenolic hydroxyl and alcoholic hydroxyl groups, are well preserved. Therefore, EHL is more suitable as a raw material for bio-production [30]. In this study, EHL was used to react with non-volatile and non-toxic glyoxal under the catalysis of sodium hydroxide solution, and the environmentally friendly wood adhesive based on the glyoxalated lignin (GEHL) was prepared. The changes between EHL and GEHL were analyzed by FTIR, TGA, GPC, DSC, and titration tests. In the whole process of the preparation of wood adhesive, there was no use of formaldehyde or phenol and tried to maximize the biomass content of adhesive. Another goal for this paper was to improve the mechanical properties of the wood adhesive by mixing it with some aqueous emulsion, such as polyacrylic ester (AE) emulsion resin and polyurethane (PU) emulsion resin, since there are a few reports of similar work in the field of lignin-based wood adhesives. 

## 2. Materials and Methods 

### 2.1. Materials

EHL was delivered by Songyuan Laihe Chemicals Co., Ltd. (Songyuan, China), and it was pre-treated at 50 °C for 24 h in a vacuum oven before using. Some parameters of lignin are listed in Table 1. Glyoxal water solution (40 wt %) was supplied by Tianjin Guangfu Fine Chemical Research Institute (Tianjin, China). Epichlorohydrin was supplied by Tianli Chemical Reagent Co., Ltd. (Tianjin, China), and the PAPI (Polymethylene polyphenyl polyisocyanate) was provided by Institute of Petrochemistry Heilongjiang Academy of Sciences (Harbin, China). Birch veneer (420 mm × 420 mm × 1.6 mm) with a moisture content of 8–12% was purchased from Jinhai Wood Co., Ltd. (Dunhua, China). The polyacrylic ester (AE) emulsion and aqueous polyurethane (PU) emulsion (industrial grade) were provided by Honghe Co., Ltd. (Dongguan, China). 

### 2.2. Preparation of the Glyoxalated Lignin 

Thirty parts EHL powder was added to 100 parts of NaOH solution with a pH of 13. This powdered EHL was soaked in water to break the lumps with vigorous stirring. Once the particles were uniformly wetted, the further dissolution was easily done. At the same time, 10 parts glyoxal solution (40 wt %) was added into the solution gradually. Ultimately, a small amount of sodium hydroxide solution (30 wt %) was dropped into the system, thus keeping the pH approximately 12.0 to improve dissolution and modification, which has been shown in previous work [31]. The reactions were carried out for 4–10 h in a 250 mL flat bottom flask equipped with a thermometer, agitator, and condenser tube, meanwhile the temperature was kept near 60 °C and stirred continuously. 

### 2.3. Preparation of the Environmentally Friendly Wood Adhesives

The GEHL solution was mixed with the epichlorohydrin uniformly, which acts as a crosslinking agent in the system. AE and PU emulsion were chosen to mix in adhesive to improve the properties of wood adhesive. By mixing all aforementioned components together thoroughly according to mass ratio, shown in *Results* section, the environmentally friendly wood adhesive was prepared, which needed to be used as soon as possible.

### 2.4. Fabrication and Bonding Strength Testing of Plywood

Initially, the birch veneers were dried in a vacuum oven with a temperature of up to 50 °C before using until the moisture content was 8–10% for each veneer. Each bonding surface was polished by 180 grit abrasive paper to improve the surface roughness of the veneer, which is beneficial to the permeability of adhesives. Three veneers were arranged alternately according to the surface texture. A thin layer of adhesive (400 g/m^2^) between the veneers was applied with the help of a brush. The stacked veneers were pre-pressed before being hot-pressed, and the hot-pressing parameters were set at a temperature of 120 °C, a pressure of 5.0 MPa, and a time of 8 min. 

The plywood was placed for more than 24 h and cut into standard samples (ten samples per group). Each sample was tensile tested by a CMT 5504 mechanical testing machine (Sans, Shenzhen, China) with a stretching speed of 5.0 mm/min, according to ASTM D2719-1989 [32]. The water resistance of the adhesives was evaluated by the wet share strength of samples, which was tested after 24 h cold water (20 °C ± 3 °C) soaking or 3 h hot water (63 °C ± 3 °C) soaking.

### 2.5. Analysis of Functional Groups for Lignin

#### 2.5.1. Titration Analysis

The total hydroxyl content of lignin samples was determined by acetylation titration [33]. The pyridine, acetic anhydride, and dioxane were mixed as an acetylated reagent with a weight ratio of 4:4.7:4.4. A mixture of lignin sample (40 mg) and acetylated reagent (0.5–0.6 g) was reacted at 50 °C for 24 h for each sample. Then, the volume of the mixture was fixed at 25 mL with the solution mixed by dioxane and water with a volume ratio of 4:1. 5 mL solution was removed and titrated with 0.1 mol/L NaOH standard solution. Ultimately, the total hydroxyl content was determined using Equation (1).

(1) $$[\mathrm{OH}]=\frac{(b_{0}-b)f\times 1.7\times 5\times 100}{A}$$

*b*_0_—The dosage of NaOH solution by blank sample, mL; *b*—The dosage of NaOH solution by sample, mL; *f*—Correction factor of 0.1 mol/L NaOH standard solution; *A*—Mass of the sample.

#### 2.5.2. Fourier Transformation Infrared Spectroscopy

The OPUS 7.5 model FTIR spectrophotometer (Bruker Optics, Karlsruhe, Germany) was used to determine the functional groups in the EHL and GEHL by an attenuated total reflection (ATR) technique. Spectra were collected from 400–4000 cm^−1^ with a resolution of 4 cm^−1^ at room temperature. 

#### 2.5.3. UV Spectrophotometer Analysis

**Figure 1:**
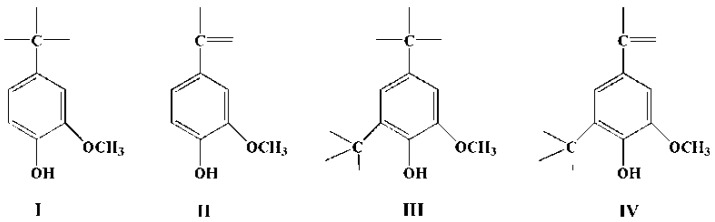
Structure of different types of phenolic hydroxyl.

Difference UV spectrophotometry is a sample method used to determine the content of phenolic hydroxyls in lignin. This method is mainly based on the dissociation of phenolic hydroxyl in alkaline solution [34,35]. A sample of 10–15 mg of lignin was weighted and dissolved in 10 mL of dioxane, and a 2 mL solution was expanded to 50 mL by using a NaH_2_PO_4_/NaOH buffer solution (pH = 6) and a 0.2 mol/L sodium hydroxide solution. Optical densities at λ (wavelength) = 300 and 360 nm were recorded with a T6 UV-vis spectrophotometer (UV) provided by Beijing Purkinje General Instrument Co., Ltd. (Beijing, China). A 0.2 mol/L NaOH solution was measured as a sample, and the buffer solution was considered a blank sample in the testing. Phenolic hydroxyl groups with different structures are shown in Figure 1.

The optical densities at 300 and 360 nm were defined as *D*′ and *D*″, and the differential extinction coefficient Δ*ε* was obtained using the following Equation (2):

(2) $$\Delta \varepsilon =\frac{D}{c\cdot l}$$

*c*—concentration of the lignin solution (g/L); *l*—cuvette thickness.

The percentage content of phenolic hydroxyl based on Equations (3)–(5) could then be found [35]:

(3) $$OH_{I+\mathrm{III}}=\frac{(\Delta \varepsilon^{\prime }+0.238\Delta \varepsilon^{\prime \prime })\cdot 1700}{4000}=0.425\Delta \varepsilon^{\prime }+0.101\Delta \varepsilon^{\prime \prime }$$

(4) $$OH_{\mathrm{II}+\mathrm{IV}}=\frac{\Delta \varepsilon^{\prime \prime }\cdot 1700}{21000}=0.081\Delta \varepsilon^{\prime \prime }$$

(5) $$OH_{I+\mathrm{II}+\mathrm{III}+\mathrm{IV}}=0.425\Delta \varepsilon^{\prime }+0.182\Delta \varepsilon^{\prime \prime }$$

### 2.6. Differential Scanning Calorimeter (DSC) Analysis

A D-204 model differential scanning calorimeter (DSC) system provided by Netzsch Scientific Instruments Co., Ltd. (Selb, Germany) was used to analyze the curing reaction of lignin with PAPI or epichlorohydrin with a mass ratio of 3:1. A 5–10 mg sample was used in the test, and the sample was heated from room temperature to approximately 300 °C with a heating rate of 10 K/min and under a nitrogen atmosphere (30 cm^2^/min). The reaction heat in the curing process for samples was defined and recorded in the DSC curves.

### 2.7. Thermogravimetry Analysis (TGA)

Approximately 5–10 mg of sample powder was weighed and tested in the instrument (Netzsch, Selb, Germany). Samples were scanned from room temperature to approximately 600 °C with a heating rate of 10 K/min and an air atmosphere, and the thermal gravity (TG) and differential thermal gravity (DTG) curves were obtained automatically during the process of warming up.

### 2.8. Molecular Weights Determination 

Before the gel permeation chromatograph (GPC) test, the lignin samples were modified by acetylation according to the method used by Glasser and Jain [36], which improved the solubility of lignin in tetrahydrofuran (THF) with almost no effect on molecular weights. In order to ensure lignin molecules disperse uniformly and stably in the solvent THF, the sample solutions (1 to 1.5 mg/mL) were stored for 24 h before testing. The number average molecular weight (*M_n_*), weight average molecular weight (*M_w_*), and molecular weight distribution (*M_w_*/*M_n_*) for EHL and GEHL were determined by an Agilent1100 model GPC, provided by Agilent Technologies Co., Ltd. (Santa Clara, CA, USA), equipped with a G1315B model UV detector and column combined with two 79911GP type columns. The test parameters were set as follows: testing temperature of 30 °C, an amount of injection of 50 μL with a flow rate of 1.0 mL/min, and a column pressure of 2.9 MPa. The columns were calibrated using polystyrene standards with different molecular weights, and the standard curve and fitting equation (*Lg*(*M*) = 10.60068 − 0.50533*t*) was obtained from the GPC system.

## 3. Results

### 3.1. Analysis of Functional Groups for Modified Lignin

#### 3.1.1. Titration Analysis

**Figure 2:**
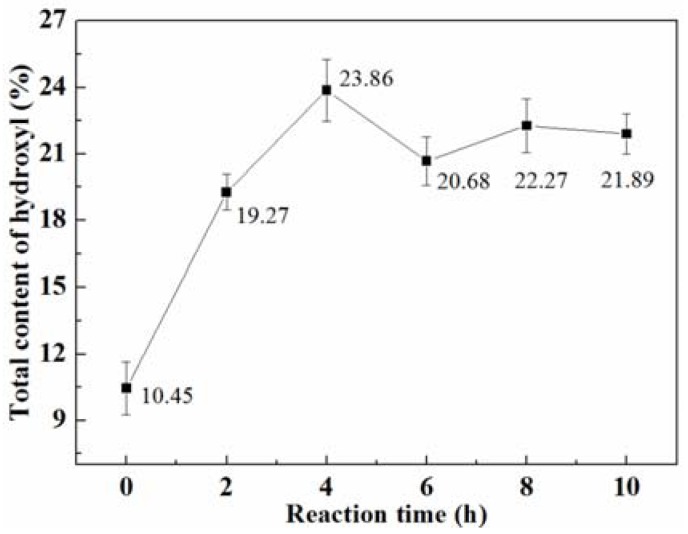
Total content of hydroxyl groups of lignin treated with glyoxal at different reaction times.

The total hydroxyl content of lignin was determined by acetylation titration and the results are shown in Figure 2. Figure 2 shows that the total hydroxyl content obviously increased after hydroxylation reaction and the maximum value of 23.86% was achieved at a reaction time of 4 h, which was more than double the original lignin total hydroxyl content. Hydroxymethyl (–CH_2_OH) and aldehyde (–CHO) groups were introduced to the active site of benzene rings in the process of the modification. Therefore, the increase of the hydroxyl groups indirectly indicated the occurrence of a glyoxalation reaction. However, there was no significant change in hydroxyl content after the 4 h reaction time. Secondary polycondensation reactions between glyoxal and lignin molecules may be responsible for the decrease in hydroxyl content.

#### 3.1.2. FTIR Analysis

**Figure 3:**
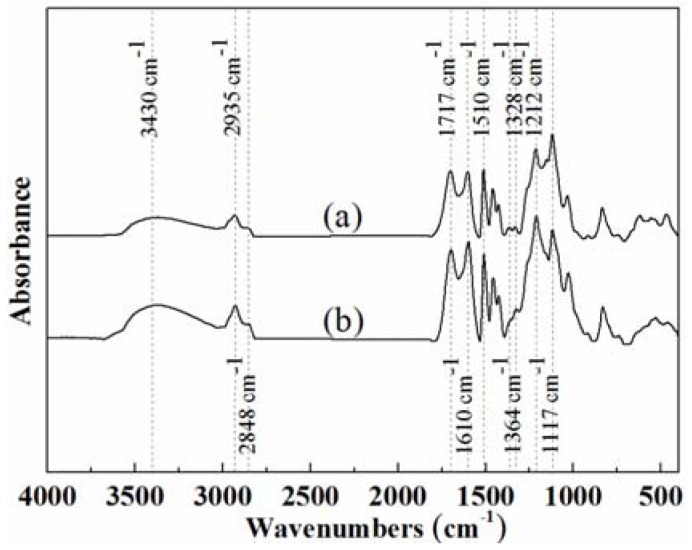
FTIR spectra of (**a**) original EHL; and (**b**) glyoxalated EHL at 4 h.

The molecular structure changes of lignin were analyzed by FTIR spectroscopy. The FTIR spectra of the original enzymatically hydrolyzed lignin and modified lignin produced at 4 h are shown in Figure 3. The attribution of characteristic absorption peaks were identified according to Faix [37]. From Figure 3, some characteristic absorption peaks can be seen clearly, such as a broad band hydroxyl absorption peak (3430 cm^−1^); the C–H stretching vibration bands (2848 cm^−1^ and 2935 cm^−1^) from methyl, methoxyl, and methylene groups; the carbonyl groups (1717 cm^−1^); phenolic hydroxyl groups (1364 cm^−1^ and 1328 cm^−1^); aliphatic hydroxyl second characteristic absorption peak (1212 cm^−1^); the ether linkage (1117 cm^−1^); and C–H vibration (1510 cm^−1^ and 1610 cm^−1^) from aromatic rings. In order to determine the content of other functional groups, the peak area at 1510 cm^−1^ was set to 1.00 [28]. Some important functional groups were identified based on Equations (6)–(8) and listed in Table 2.

The value of total OH-group = (*A*_3430_ + *A*_1364_ + *A*_1328_ + *A*_1212_)/*A*_1510_ (6)

The value of phenolic OH-group = (*A*_1364_ + *A*_1328_)/*A*_1510_ (7)

The value of aliphatic OH-group = (*A*_3430_ + *A*_1212_)/*A*_1510_ (8)

where *A_k_* refers to the absorption peak at wavenumber *k*.

Figure 3 shows that the same groups were present in EHL and GEHL, but the content changed. Table 2 summarizes the ratios of relative absorbance for different functional groups from lignin modified with glyoxal at different times. Compared with original EHL, there was a significant increase in the content of OH-aliphatic and OH-total groups after the hydroxyethylation reaction. The peak intensity at 1212 cm^−1^ increases from 2.45 to 2.86 gradually with the extension of reaction time. This increase can be explained by noting that the hydroxyl (–CH_2_OH) and aldehyde (–CHO) groups were introduced into lignin molecules and the amount of introduction increased gradually. On the other hand, the content of aromatic hydroxyl groups remained constant (1.8 to 2.0) during the first 6 h and then decreased slightly. At the same time, the content of the alcoholic hydroxyl decreased significantly after it reached the maximum value of 7.58. This may be caused by the side reaction, which was a secondary polycondensation reaction [26].

As a component of wood adhesive, lignin can be used to prepare lignin-based polycondensate resins by reacting with aldehyde, phenol, isocyanate, or other biomass resources, e.g., tannin, under heating conditions. However, a high content of hydroxyl is necessary, which could be found in modified lignin that reached the maximum value of 7.78 at a reaction time of 4 h.

#### 3.1.3. UV Spectrophotometer Analysis

The content of phenolic hydroxyl with different structures were decided by calculation based on optical density (D) and summarized in Table 3. The changes of structural characteristics in the process of reaction were mainly carried out on free C-3 and C-5, so the lignins of type I and II are more active than other types. At the same time, the lignin of type II may be more active than type I because an electron withdrawing group (–C=O) reduced the electron cloud density of benzene ring, which is beneficial to the occurrence of electrophilic reactions. From Table 3, it can be seen that the total phenolic hydroxyl content in EHL molecules is about 3.5, 88% of which are types I and III. Only 12% are types II and IV phenolic hydroxyl groups in lignin molecules. It is clear that types I, II, III and IV decreased simultaneously with extension of reaction time. At the same time, the decrease after 8 h is more obvious than other reaction periods. The trend is similar to the results from FTIR analysis, which may be caused by secondary polycondensation reactions between lignin fragments, or lignin and glyoxal. Phenolic group has a high reactivity with lignin molecules, so if the content reduced significantly, this will be detrimental to the improvement of lignin activity.

### 3.2. DSC Analysis

**Figure 4:**
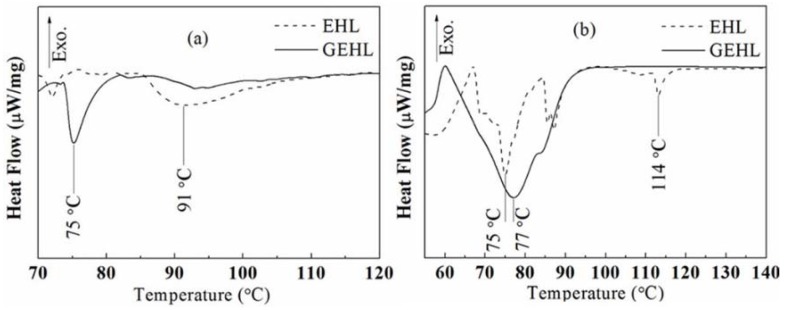
The reaction DSC curves of (**a**) lignin and PAPI, and (**b**) lignin and epichlorohydrin.

There were some hydroxyl groups in the EHL or GEHL molecules which can react with isocyanate groups (–NCO) or epoxy group and crosslink a network structure. The reaction heat was determined by DSC, which can reflect the reactivity of lignin samples indirectly. The mixed sample of EHL and PAPI, and EHL and epichlorohydrin was tested as a reference. Another sample was mixed using PAPI or epichlorohydrin and the GEHL with a reaction time of 4 h. Both of the DSC curves are shown in Figure 4 and some important parameters were marked.

From Figure 4a, there is no obvious endothermic or exothermic peak in the DSC curve of EHL/PAPI sample. Only a small broad endothermic peak is found at 91.0 °C, which starts at 86.0 °C and has an offset temperature at 106 °C. Compared with EHL/PAPI sample, the sample of GEHL/PAPI shows a strong and narrow endothermic peak at 75.0 °C. From Figure 4b, the reaction peak from sample GEHL/epichlorohydrin is also stronger than EHL/epichlorohydrin reaction peak. Furthermore, there is an endothermic peak at 114 °C for the EHL/epichlorohydrin curve which can be explained by noting that a small amount of epichlorohydrin did not participate in the reaction and remained in the sample. On the one hand, due to the macromolecule aggregation effect, the hydroxyl groups were wrapped inside the particle of lignin, which prevented the reaction with the isocyanate group. On the other hand, the limited amount of hydroxyl groups on the original EHL may be another reason for the low reactivity. During the process of liquefaction and modification, the content of hydroxyl groups increased with the introduction of –CH_2_OH and the macromolecule aggregation effect was destroyed under the conditions of a strong base and heating.

From the analysis of DSC curves, it is clear that the reactivity of lignin increased significantly. This is not limited to isocyanate-based or epichlorohydrin curing agents, since it is also common to other curing agents that can react with active hydroxyl, such as epoxy and aldehydes. The increase of the reactivity is important for its application in adhesives or other biomass materials.

### 3.3. Thermogravimetric Analysis

**Figure 5:**
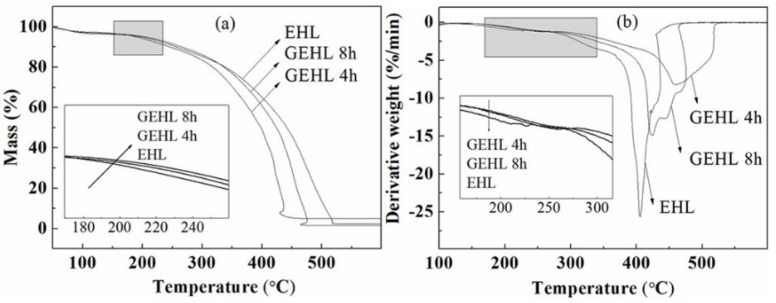
(**a**) TG and (**b**) DTG curves of EHL and GEHL.

Figure 5 shows the (a) TG and (b) DTG curves of EHL and GEHL (GEHL4 and GEHL8) prepared using different reaction times. Some important parameters are summarized in Table 4. From the TG curves, there was a slight loss of mass for all lignin samples before 120 °C, which may be caused by evaporation of moisture or other small volatile molecules. At this stage, the mass loss rate (%/min) for all samples is between 2.5%/min and 3.5%/min. This phenomenon has also been reported by other scholars [38].

For most lignocellulosic materials, including our samples, the initial thermal decomposition temperature is generally between 200 to 250 °C. At the same time, there was a similar degradation trend for all samples, but the EHL degrades first, and the difference in the weight loss of EHL and GEHL subsequently became evident. From the DTG curves, it is clear that the maximum decomposition rate reduced after glyoxalated, and down to 8.2%/min at a reaction time of 4 h. Meanwhile, the peak of DTG_max_ clearly moves to a high temperature after modification. All of these indicate that the thermal performance of lignins improved with the hydroxyethylation, which can be explained by the molecular weight of lignin increasing because there may be a polycondensation reaction between lignin and glyoxal molecules during the reaction. It can be seen from the DTG curves that the derivative weight of the GEHL shows a gradual decrease, which can clearly be seen from the sample of GEHL8. This phenomenon may be caused by the difference of reaction degree for hydroxyethylation or polycondensation with glyoxal molecules. Multimers could have also formed by self-condensation of small molecules, which may be another reason for the phenomenon, for example, the glyoxal which was not involved in the reaction. The residue for GEHL samples at the end temperature of 600 °C was between 0.5 and 3.0%, which was significantly lower than original EHL with a residue of 5.2%. The increase of free volume within the molecule led it to thermally degrade more fully, which may be caused by the growth of a side chain on C-3 and C-5 positions with the introduction of the −CH_2_OH and −CH_2_CHO groups [36]. 

From the results of TGA, the GEHL prepared over 4 h of reaction time presented the better thermal stability, and was more suitable as a raw material for adhesive formulation or other composite biomass applications.

### 3.4. Molecular Weights Determination

**Figure 6:**
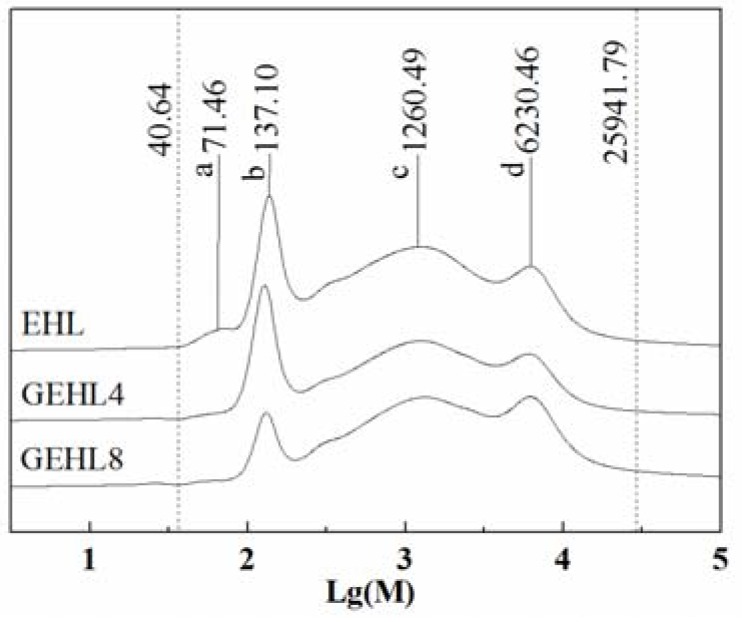
The GPC curves of EHL and GEHL.

The GPC curves of EHL and GEHL over a reaction time of 4 h and 8 h are shown in Figure 6. Moreover, several major peaks, and the corresponding molecular weights, are marked as *a*, *b*, *c,* and *d* in the figure. The EHL has a wide molecular weight distribution from 40.64 to 25,941.79 g/mol. There are four main peaks in the GPC curves of lignin samples, which indicate that the molecular weight of EHL is mainly concentrated in four regions, 71.46, 137.10, 1260.49, and 6230.46 g/mol. From Figure 6, the *a* peak of the GEHL is seen to disappear between samples. The low molecular weight lignin molecule at peak *a* has the strongest reactivity in all lignin molecules. Furthermore, this part of the lignin molecule reacted with glyoxal at first, and when the glyoxal was introduced in lignin, the molecular weight of the modified lignin increased to approximately 128 g/mol, and the peak combined with the original molecular peak at 137.10. All of the above explained the left shift and increase of peak *b* for GEHL4 sample. For the sample of GEHL8, the *b* peak decreased significantly and the *c* and *d* peaks slightly shifted to the right, which can be explained by the presence of a secondary polycondensation reaction between lignin molecules and glyoxal or self-condensation reaction of lignin during the long reaction time.

Number-average (*M_n_*) and weight-average (*M_w_*) molecular weights, and polydispersity (defined by the ratio *M_w_*/*M_n_*) of all lignin samples are shown in Table 5. Compared with the original lignin, the *M_n_* and *M_w_* increased gradually, but the change is not obvious during the reaction time between 0 and 4 h. These minor changes may be due to the introduction of the glyoxal. After the samples had reacted for 4 h, the *M_n_* and *M_w_* increased significantly with the gradual polymerization of active lignin molecule fragments. It was reported that such a polycondensation reaction can easily happen between lignin fragments and some α-hydroxyl groups under the catalysis of an alkaline condition, and form an alkali-stable methylene linkage eventually [39]. Not only that, the polydispersity decreased after the glyoxalation reaction and the trend enhanced as the reaction time increased.

### 3.5. The Mechanical Properties of Plywood

**Figure 7:**
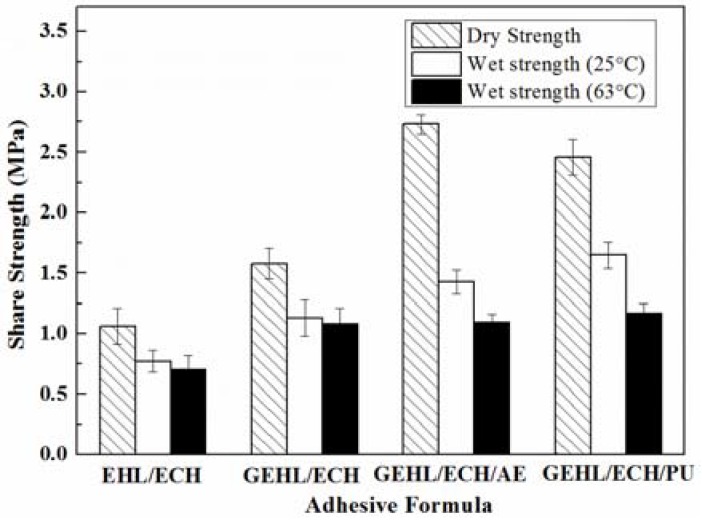
Shear strength and water resistance of lignin-based adhesives: EHL:ECH = 100:30 (*w*/*w*); GEHL:ECH = 100:30 (*w*/*w*); GEHL:ECH:AE = 100:20:15 (*w*/*w*/*w*); GEHL:ECH:PU = 100:20:15 (*w*/*w*/*w*).

Epichlorohydrin can react with lignin and form a cross-linked network, which is the source of adhesive bond strength. Dry strength and wet strength of plywood based on lignin-based wood adhesives are shown in Figure 7. From the comparison between EHL/ECH and GEHL/ECH, it is clear that dry strength and wet strength of adhesive from GEHL was significantly higher than from EHL, which reached 1.58 MPa (dry strength) and 1.08 MPa (63 °C wet strength) for GEHL/ECH. These results indicate that the content of active groups and reactivity for GEHL increase significantly with the introduction of glyoxal. 

Although the adhesive from GEHL/ECH was successfully applied to the preparation of plywood, some problems still existed, such as the large dosage for ECH and the low quality of mechanical properties. GEHL/ECH/PA and GEHL/ECH/PU were prepared by adding a small amount of AE emulsion and PU emulsion into the GEHL/ECH system. The dry shear strength significantly increased when the emulsion was mixed into the adhesive, which went up to 2.73 MPa for GEHL/ECH/AE and 2.46 MPa for GEHL/ECH/PU; both wet shear strengths are above 1.0 MPa too. At the same time, the dosage of ECH decreased without affecting mechanical performance with the addition of emulsion. In the hot-pressing process of plywood, complex physical and chemical reactions occurred between the various active groups in emulsion, lignin, ECH, and wood.

## 4. Conclusions

The performance of lignin was improved by the hydroxyethylation reaction between corn stalk enzymatic hydrolyzed lignin and glyoxal. Active groups were introduced into the molecule of lignin using a hydroxylation reaction, such as alcohol hydroxyl and an aldehyde group. Compared with original lignin, the reactivity between modified lignin and isocyanates or epoxy curing agents was significantly enhanced. In the process of modification, the thermal performance of lignin molecules increased, and at the same, the molecular weight changed too, but the change was not obvious within a 4 h reaction time. Environmental wood adhesives based on glyoxalated lignin were prepared and plywood with good mechanical properties (e.g., dry strength 2.73 MPa) was obtained. Compared to the original lignin, the modified lignin was more reactive and can effectively be applied to wood adhesives or other composite biomass as raw material.

## Figures and Tables

**Table 1:** Properties of the lignin.

Properties	Moisture Content/%	Ash Content/%	pH	Purity/%	Average Particle Size/μm	*T*_g_/°C	Hydroxyl Content/%	Carboxyl Content/%
Results	3.35	4.49	4.59	90 *	11.35	181	10.45	2.12

* Parameters provided by the manufacturer.

**Table 2:** Ratios of relative absorbance for different functional groups.

Samples	Modification Time (h)	OH-Aromatic	OH-Aliphatic	*A*_1212_/*A*_1510_	OH-Total
EHL	0	0.20	5.58	1.46	5.78
GEHL2	2	0.18	6.93	2.45	7.11
GEHL4	4	0.20	7.58	2.44	7.78
GEHL6	6	0.20	6.76	2.41	6.96
GEHL8	8	0.19	6.54	2.52	6.73
GEHL10	10	0.19	6.35	2.86	6.54

**Table 3:** The content of phenolic group in lignin samples.

Samples	EHL	GEHL2	GEHL4	GEHL6	GEHL8	GEHL10
OH_I+III_	3.141	2.991	2.987	2.970	2.939	2.810
OH_II+IV_	0.437	0.430	0.426	0.390	0.405	0.400
OH_total_	3.578	3.421	3.413	3.360	3.344	3.210

**Table 4:** Results from thermal analysis for *T*_i_, DTG_max_, *T*_end_, and residue (%).

Sample	Modification Time (h)	*T*_i_ (°C)	DTG_max_ (°C)	*T*_end_ (°C)	Maximum Decomposition Rate (%/min)	Residue (%)
EHL	0	220.7	405.8	455.8	25.6	5.2
GEHL2	2	243.3	439.1	515.1	14.7	2.8
GEHL4	4	241.1	459.8	527.7	8.2	2.5
GEHL6	6	245.3	447.2	501.3	10.4	2.2
GEHL8	8	244.7	423.5	471.0	14.9	1.7
GEHL10	10	244.8	409.4	489.4	15.2	0.5

**Table 5:** Results of *M_n_*, *M_w_*, and polydispersity of acetylated lignin samples.

Samples	Modification Time (h)	*M_n_* (g/mol)	*M_w_* (g/mol)	*M_w_*/*M_n_*
EHL	-	371	2295	6.18
GEHL2	2	377	2299	6.09
GEHL4	4	386	2232	5.77
GEHL6	6	593	2968	5.01
GEHL8	8	613	3048	4.97
GEHL10	10	523	2900	5.55
